# Simulation study of oxytetracycline contamination remediation in groundwater circulation wells enhanced by nano-calcium peroxide and ozone

**DOI:** 10.1038/s41598-023-36310-1

**Published:** 2023-06-05

**Authors:** Xinyi Wang, Lei Zhang, Chunmei Han, Yanyan Zhang, Jiaxin Zhuo

**Affiliations:** 1 School of Earth Science, Northeast Petroleum University, Daqing, 163319 Heilongjiang China; 2 The Third Oil Plant of Daqing Oilfield Co.Ltd.Daqing, Daqing, 163113 Heilongjiang China; 3 Shandong Academy of Environmental Science Environmental Testing Co., Ltd., Jinan, 250013 Shandong China

**Keywords:** Environmental sciences, Environmental chemistry, Geochemistry

## Abstract

The widespread use of antibiotics in recent years has led to increasing antibiotic contamination of shallow groundwater. As the most widely used tetracycline antibiotic, oxytetracycline has received a lot of attention from researchers due to its stable molecular structure and difficulty in degradation. Aiming at remediation of oxytetracycline pollution in shallow groundwater, nano-calcium peroxide (nCaO_2_) and ozone (O_3_) are used to enhance the degradation of oxytetracycline in groundwater circulation well (GCW). A three-dimensional sand box test device for circulation wells is designed to explore the repair efficiency of circulation wells strengthened by different oxidants. The results show that after nCaO_2_ and O_3_ enhancing circulation wells operate for 10 h, the average removal rate of OTC reaches 83%, and the highest removal rate is 88.13%, which is 79.23% and 13.96% respectively higher than that of nCaO_2_ and O_3_ enhanced circulation wells alone, and there is no rebound phenomenon after aeration stops. The in-situ treatment of enhanced GCW by nCaO_2_ and O_3_ has potential applications for the removal of OTC in groundwater environments.

## Introduction

Oxytetracycline is the most common tetracycline antibiotic^1^. It is widely used in animal husbandry, but only a small amount of oxytetracycline is absorbed and generally enters the environment in the form of faeces. Oxytetracycline has a stable molecular structure, which is difficult to be degraded by microorganisms, and it exists for a long time and causes environmental pollution^2–5^. López-Serna et al.^6^ detected the antibiotic concentration in the groundwater of Barcelona, Spain, and found that the local groundwater was polluted to varying degrees, with the highest concentration of tetracycline reaching 188 ng/L. Jiang^7^ detected 16 antibiotics in groundwater samples in northern China, and found that the content of oxytetracycline in groundwater in this area was 8325.8 ng/L. Therefore, at present, controlling oxytetracycline pollution is the primary task to eliminate antibiotic pollution^8^.

To address oxytetracycline contamination in groundwater, there are two common approaches: in-situ remediation and ex-situ remediation. Because antibiotics flow with water when they enter groundwater, ex-situ remediation is costly and the contaminant cannot be treated for a long time, so one of the in-situ remediation methods is chosen to treat the contamination. Groundwater Circulation Well (GCW) technology is based on in-situ air disturbance technology and gas phase extraction technology^9,10^. However, GCW technology alone can only transfer pollutants to the ground for treatment and cannot fundamentally degrade pollutants directly. Therefore, other means are often used to strengthen GCW, such as biological reinforcement^11,12^, electrical reinforcement^13^ and surfactant reinforcement^14,15^. Ozone and calcium peroxide can be used to remove organic pollutants from groundwater. Ozone has the advantages of fast reaction speed and remarkable effect, and is widely used in groundwater treatment^16–18^, but it is easy to cause pollutants to rebound. As an oxidant, calcium peroxide will also produce O_2_ when it decomposes organic matter, which can provide oxygen source for microorganisms and remove pollutants faster^19,20^, so it is widely used in groundwater remediation. At the same time, the research shows that Ca(OH)_2_, another hydrolysis product of CaO_2_, can adsorb pollutants, and it is not easy to cause pollutants to rebound^21,22^.

As a highly active oxidant, nano-calcium peroxide can rapidly oxidize and decompose harmful substances such as oxytetracycline. Ozone is a strong oxidizing substance that can accelerate the decomposition and removal of oxytetracycline, thereby quickly and effectively repairing contaminated groundwater. NCaO_2_ and ozone are non-toxic and harmless environmental protection materials, which will not cause secondary pollution to the environment, and are more in line with the concept of green environmental protection development.Through indoor three-dimensional simulation experiments, the author explored the in-situ remediation of oxytetracycline contaminated groundwater by nCaO_2_ and O_3_ enhanced circulation wells, optimized the operating parameters of circulation wells, and put forward new ideas for long-term remediation of antibiotic contaminated sites.

## Experimental materials and methods

### Experimental reagents and instruments

Oxytetracycline, sodium hydroxide, ammonia, cetyl trimethyl ammonium bromide (CTMAB), hydrogen peroxide, calcium chloride, anhydrous ethanol.

752N UV–Vis spectrophotometer: Shanghai Precision Scientific Instruments Co., Ltd.; IZ15 peristaltic pump: Baoding Lange Constant Flow Pump Co., Ltd.; SK-CFG-10P ozone generator: Jinan Sankang Environmental Protection Technology Co., Ltd.; FA1004N electronic analytical balance: Shanghai Precision Scientific Instrument Co., Ltd.; pHS-3B Precision Acidimeter: Shanghai Jiangyi Instrument Co., Ltd.; DGG-9053A electrothermal constant temperature blast drying oven: Shanghai Precision Scientific Instrument Co., Ltd.; Three-dimensional simulation sand box.

### Experimental device

**Figure 1:**
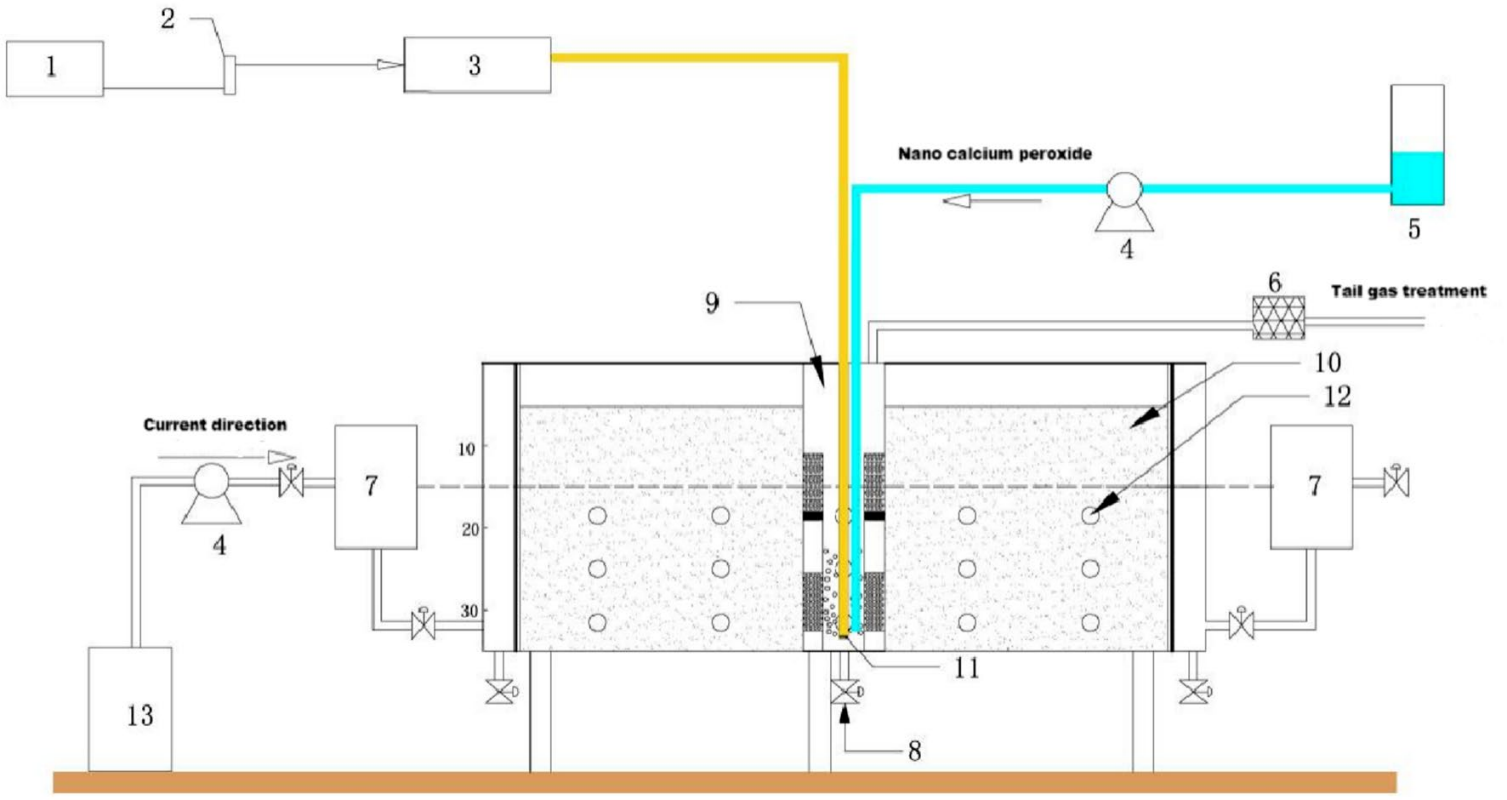
Simulation box experimental device, (1-oxygen generator; 2- flowmeter; 3- ozone generator; 4- peristaltic pump; 5- nCaO_2_ solution; 6- activated carbon; 7- water tank; 8- water injection port; 9- circulation well; 10- quartz sand; 11- aeration port; 12- sampling port; 13- distilled water tank).

The size of the three-dimensional simulation box is 80 cm × 30 cm × 35 cm, as shown in Fig. 1,with a total of 15 sampling ports, to facilitate experimental sampling.The middle position of the circulation well is connected with the ozone generator, and a small hole is opened at the side to make the calcium peroxide solution flow into the circulation well, so as to ensure the contact between ozone and calcium peroxide to assist the rapid diffusion of the solution.

The quartz sand was washed with a large amount of water after passing through a 2 mm sieve, and then put into a blast drying box above 100 °C for 5 h for disinfection and sterilization. After cooling, the experimental device was filled to 5 cm above the sand box. The local tap water is used to simulate groundwater, and the peristaltic pump is used to slowly inject water into the left small tank at a flow rate of 8 cm/d to simulate groundwater flow. The experimental device uses quartz sand to simulate the aquifer, and the specific parameters of quartz sand are shown in Table 1.

**Table 1:** Physical parameters of quartz sand.

Physical parameters	Grain size (mm)	Osmotic coefficientc (cm/s)	pH	Poriness	Density (g/cm^3^)	Permeability (m^2^)
Number	1–2	0.005	7.73	0.42	2.06	2.59 × 10^–11^

### Reaction principle

Calcium peroxide combined with ozone technology is widely used to remove organic pollutants in groundwater and pretreat high-concentration organic wastewater. Calcium peroxide can catalyze ozone to produce more OH·, enhance the oxidation capacity of the system (Formulas 1–6), and produce O_2_, which can provide oxygen source for microorganisms and remove pollutants faster^19^. In addition, researchers have shown that Ca(OH)_2_, another hydrolysis product of CaO_2_, can adsorb pollutants and achieve the purpose of degrading pollutants^23,24^.

1 $${\text{CaO}}_{2} + {\text{2H}}_{2} {\text{O}} \to {\text{H}}_{2} {\text{O}}_{2} + {\text{Ca}}\left( {{\text{OH}}} \right)_{2}$$

2 $${\text{O}}_{3} + {\text{OH}}^{ - } \to {\text{HO}}{}_{2}^{ - } + {\text{O}}_{{2}}$$

3 $${\text{H}}_{{2}} {\text{O}}_{2} + {\text{H}}_{{2}} {\text{O}} \to {\text{HO}}_{2}^{ - } + {\text{H}}_{{3}} {\text{O}}^{ + }$$

4 $${\text{O}}_{3} + {\text{HO}}_{{2}}^{ - } \to {\text{OH}} \cdot + {\text{O}}_{{2}}^{ - } + {\text{O}}_{{2}}$$

5 $${\text{O}}_{{3}} + {\text{O}}_{{2}}^{ - } \to {\text{O}}_{{3}}^{ - } + {\text{O}}_{{2}}$$

6 $${\text{O}}_{{3}}^{ - } + {\text{H}}_{{2}} {\text{O}} \to {\text{OH}} \cdot + {\text{O}}_{{2}} + {\text{OH}}^{ - }$$

### Material preparation

According to the method of literature ^25^, 4 g CaCl_2_ was added to 40 ml distilled water and stirred evenly, and 0.6 g CTMAB was mixed with 160 ml distilled water as dispersant. The two solutions were mixed and transferred to a magnetic stirrer. After mixing evenly, the pH was adjusted to about 10. The continuous dripping of 15 mL 28% hydrogen peroxide solution kept the rate stable and adjusted the pH to about 11. At this time, White is slowly produced in the solution color precipitate, stand for 3 h.The upper layer of the beaker solution was filtered, the lower layer of the precipitate is centrifuged at 3000 rpm, the lower precipitate is washed with anhydrous ethanol, and the resulting mixed solution is placed in a suction filter for suction filtration. The filter cake was dried in a vacuum drying box at 60℃. NCaO_2_ can be obtained after full grinding of the obtained powder.

### Experimental methods

#### Material characterization

Scanning Electron Microscope (SEM) scans the surface of the sample by high-energy electron beam and converts it into the surface shape of the sample by secondary electron signal imaging. X-ray diffraction is to determine the crystal structure and material phase.

#### Optimisation of reaction conditions

A beaker experiment was conducted to investigate the effect of different factors on the degradation of oxytetracycline by nCaO_2_/O_3_ system, such as the concentration of oxidation, the concentration of starting oxytetracycline and the pH of the solution.

#### Different oxidants strengthen the groundwater circulation well

After 35 days of simulated contaminant leakage, the oxytetracycline leakage experiment was stopped and stood for 20 h. The GCW remediation of the OTC experiment was performed with different oxidants under optimal reaction conditions. Intermittent aeration was used in the experiment, with a total of 5 aerations, ozone aeration for 2 h, followed by closure of the ozone generator and standing for 1 h. When the water level in the sand box is stable, samples are taken from each sampling port and the OTC concentration in the groundwater is detected. Concentration contour maps were drawn and the remediation efficiency was analyzed.

## Results and discussion

### Material characterization

#### The morphological structure of the nCaO_2_

**Figure 2:**
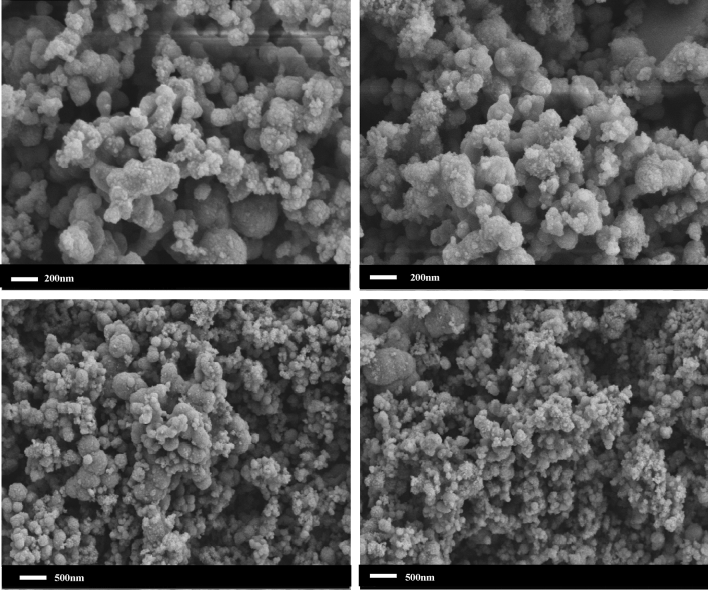
The SEM image of the nCaO_2_.

The SEM image of nCaO_2_ is shown in Fig. 2. The particle size and morphology of the nanoparticles can be clearly observed by electron microscope images.

Calcium chloride and hydrogen peroxide are used as the main materials, and CTMAB is used as the dispersant. The characterization results show that when the average diameter of the prepared nanoparticles is between 80 and 150 nm, nCaO_2_ accords with the particle size of nano-sized materials, and the particles are uniformly dispersed and there is no agglomeration.

#### Analysis of the main components of nCaO_2_

**Figure 3:**
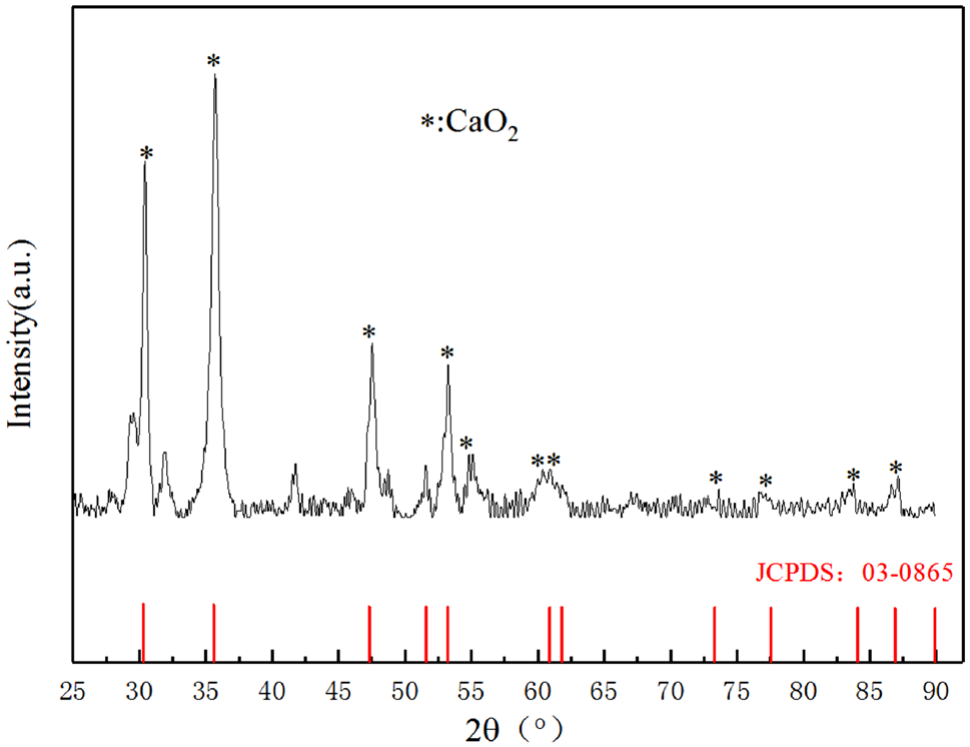
XRD image of nCaO_2_.

Whether the material is CaO_2_ can be qualitatively prepared by X-ray diffraction (XRD), and the effect of the added dispersant on the main structure and composition of the particles can be analyzed. It can be confirmed by Fig. 3 that there are characteristic peaks at 2θ of 30.4°, 35.73°, 47.48°, 51.56°, 53.24°, 60.89°and 61.78°. Compared with the standard card of CaO_2_, it is found that the positions of the diffraction peaks are the same, indicating that the main components are the same. The standard line is not a smooth straight line, indicating that there are a small amount of impurities in the prepared material, but it will not affect the properties of nCaO_2_.

### Optimization of degradation conditions

By varying one variable at a time in beaker experiments, it was concluded that a starting concentration of 20 mg/L of Oxytetracycline, 0.25 g/L of calcium peroxide solution and 8 mg/L of O_3_ at laboratory room temperature, with the solution pH on the alkaline side, was the most effective in degrading Oxytetracycline.

### Strengthen groundwater circulation wells to repair OTC contaminated groundwater

#### NCaO_2_-assisted GCW experimental study

**Figure 4:**
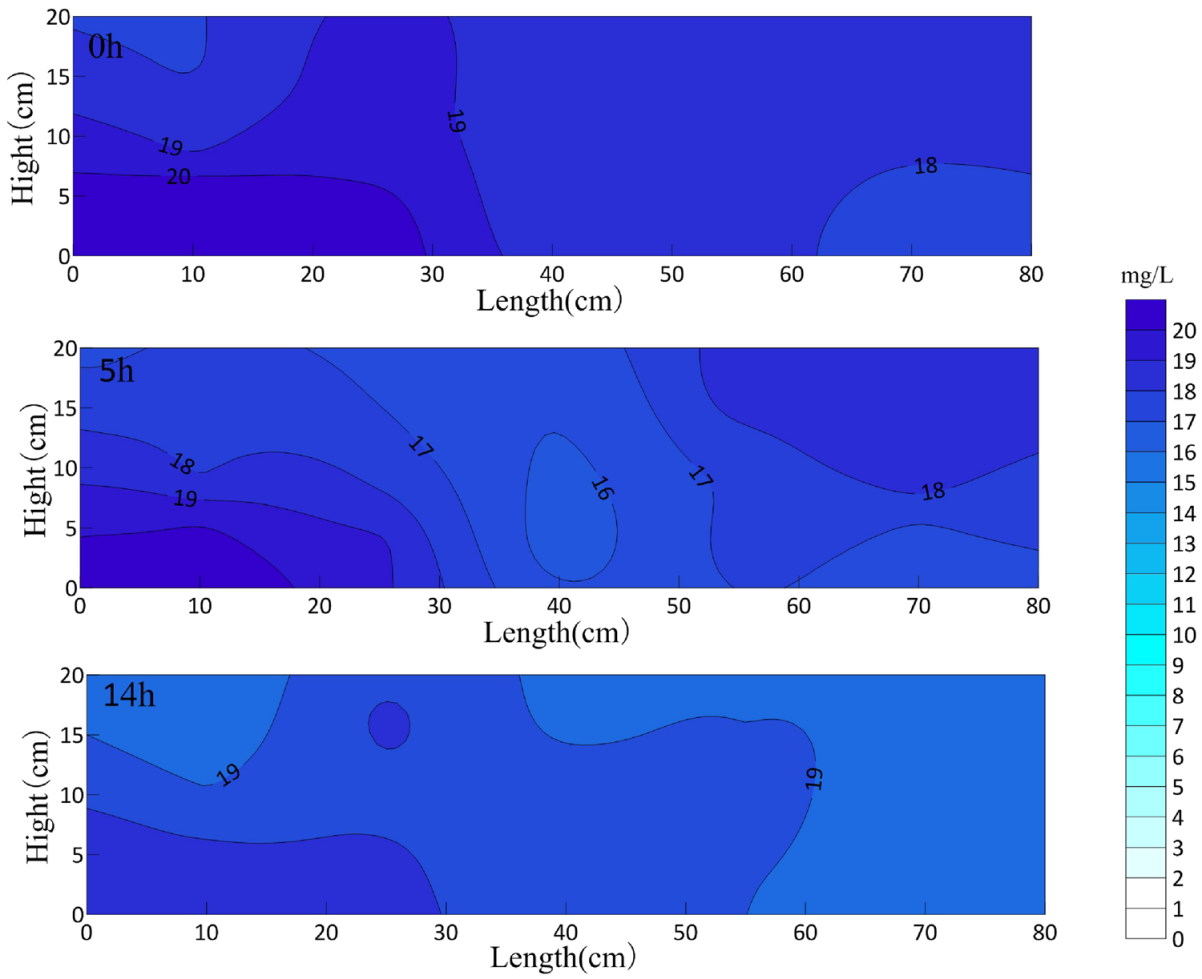
OTC concentration distribution in sand box with different aeration time (mg/L).

The OTC concentration in the sand box at different aeration times is shown in Fig. 4. The horizontal axis is the length of the sand box, and the vertical axis is the height from the bottom of the sand box. The circulation well is installed at the center line of the sand box.

After 14 h of aeration, the average concentration of oxytetracycline in the sand box decreased from the initial 17.35 mg/L to 15.72 mg/L, and the degradation rate was only 8.9%. The possible reason is that nCaO_2_ is difficult to dissolve in water, and the granular media in the aquifer will affect the diffusion of nCaO_2_, even the pores of the media will absorb a small amount of nano-calcium peroxide particles, and the oxytetracycline itself has a stable structure. However, under the influence of aeration, nano-calcium peroxide in aquifer flows into the whole box with the circulation of water, and oxytetracycline is distributed more evenly in the sand box under the impetus of circulation.

#### O_3_-assisted GCW experimental study

With the increase of aeration time, the OTC concentration in the whole sand box decreases significantly. From a horizontal point of view, the closer to the circulation well, the higher the OTC degradation efficiency, and vice versa. From the vertical point of view, OTC in the upper part of GCW is degraded first, then in the middle part, and the lowest efficiency is in the lower part of the circulation well. It can be clearly seen that the repairing area of the circulation well is an approximate conical area with the centerline of GCW as the axis, and the oxytetracycline concentration in the whole box is symmetrically distributed.

**Figure 5:**
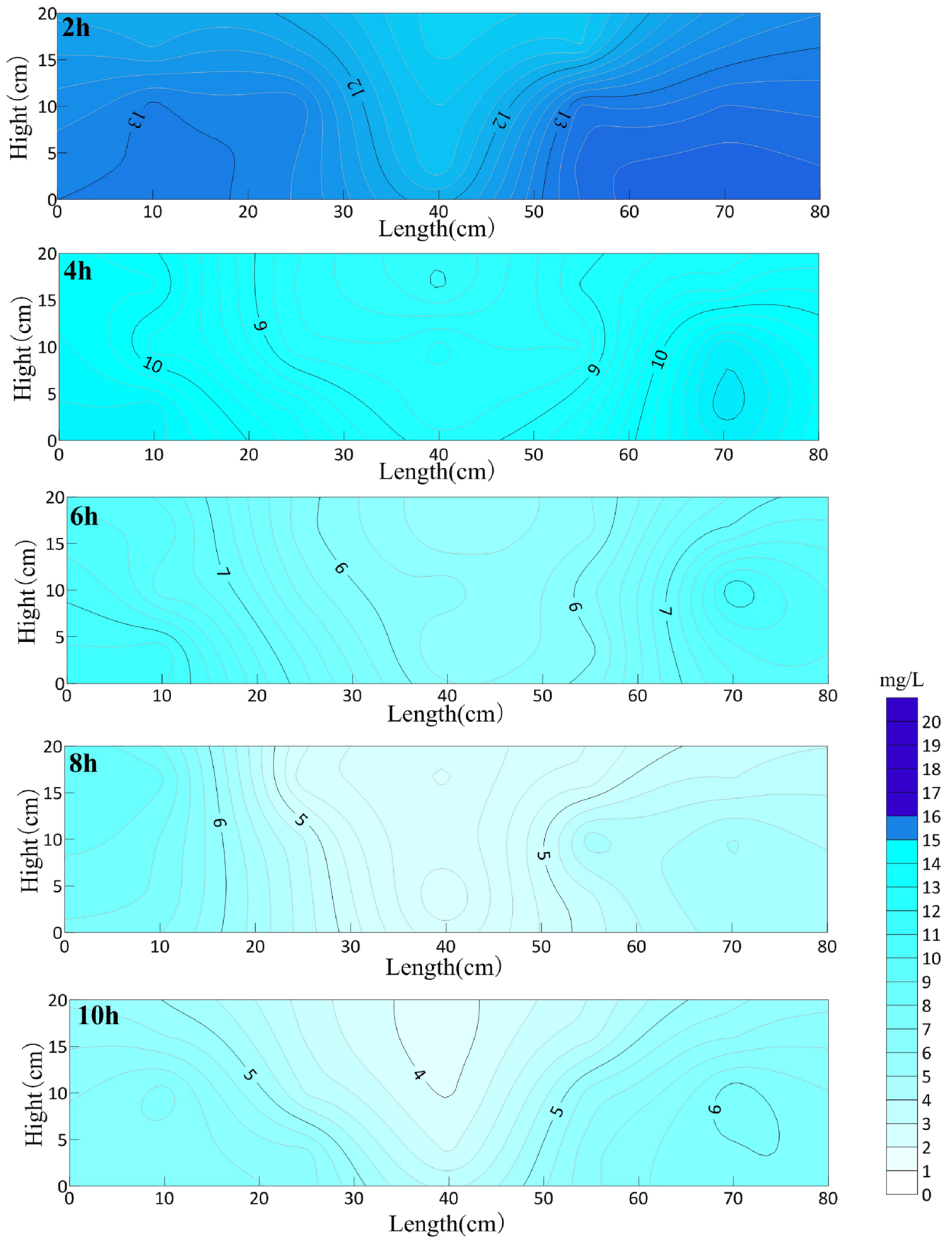
OTC concentration distribution in sand box with different aeration time (mg/L).

Figure 5 shows the distribution of oxytetracycline in the sand box at different aeration times. It can be seen from the figure that the average concentration of oxytetracycline decreased to 12.62 mg/L in the first 2 h of the reaction, which decreased by 36.8% compared with that before aeration, and the removal rate was 0.061 mg/min. With the aeration experiment, the removal rate of oxytetracycline decreased slowly within 2–10 h of cumulative aeration, and after 10 h of cumulative aeration, the average concentration of oxytetracycline will reach 5.16 mg/L, which is 74.17% lower than that before aeration. After aeration, the tailing concentration of oxytetracycline is 5.16 mg/L.

#### Experimental study on GCW enhanced by nCaO_2_and O_3_

**Figure 6:**
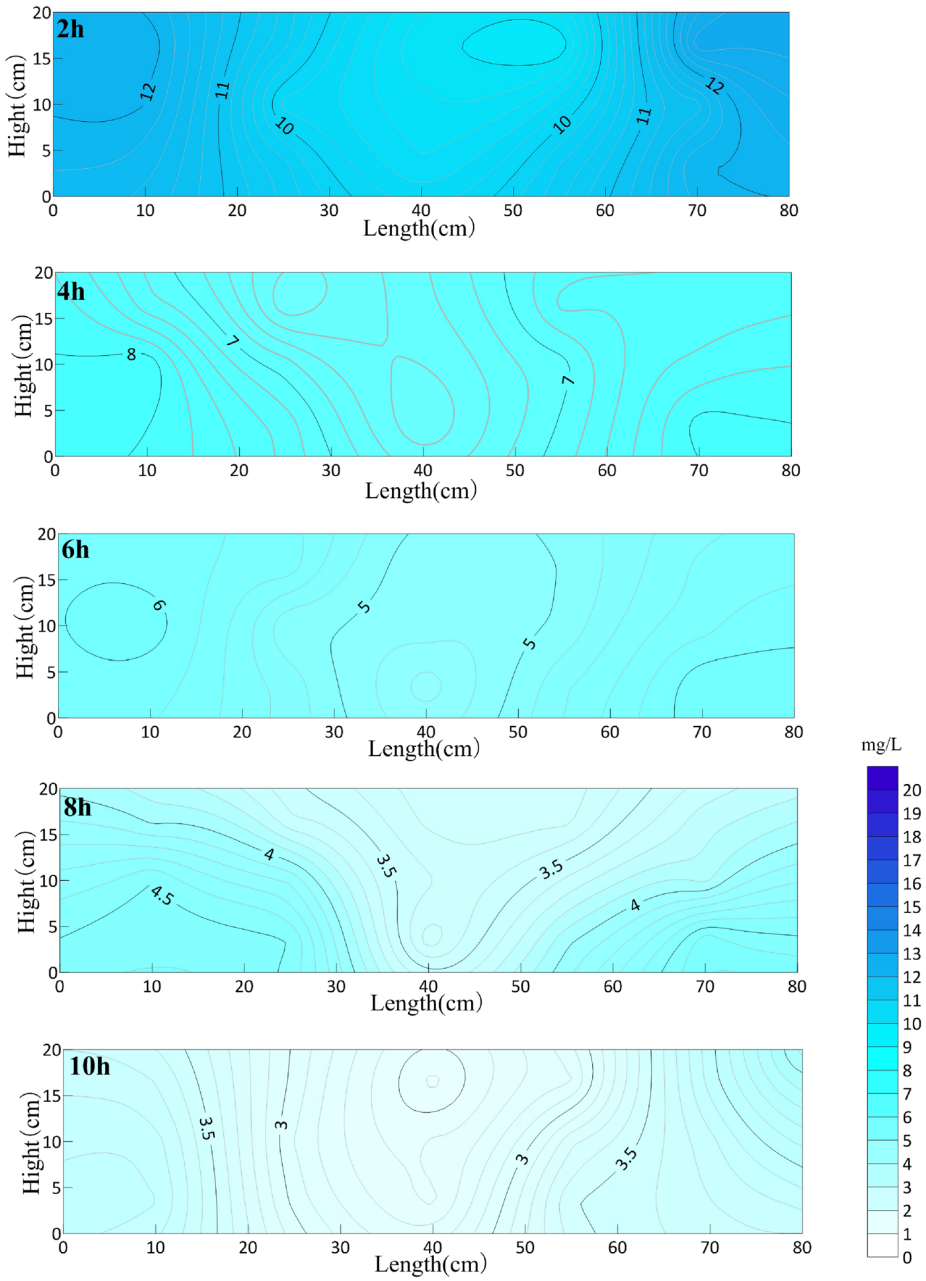
OTC concentration distribution in sand box with different aeration time (mg/L).

As can be seen from Fig. 6, with the continuous aeration experiment, the concentration of oxytetracycline near the circulation well decreases the fastest, and it presents a symmetrical distribution centered on the aeration pipe in the circulation well. The concentration of OTC in the sand box decreases gradually, but the degradation efficiency is the highest in the area close to the circulation well, and the removal efficiency is the slowest in the area far away from the circulation well, that is, on both sides of the sand box. In the first 2 h of the reaction, the average concentration of oxytetracycline decreased to 10.93 mg/L, which was 45.35% lower than that before aeration, and the removal rate was 0.076 mg/min. With the aeration experiment, the removal rate of oxytetracycline decreased slowly. After 10 h of aeration, the average concentration of oxytetracycline reached 3.4 mg/L, which was 83% lower than that before aeration. After aeration, the remediation entered the tailing stage, and the tailing concentration of oxytetracycline was 3.4 mg/L. Oxytetracycline is a refractory organic substance, and the presence of media with different particle sizes in the aquifer will hinder the contact between O_3_ and pollutants, thus affecting the oxidation efficiency. Through the analysis of the experimental results, when circulation well technology is used to remediate refractory organic substances, it will achieve better remediation effect when combined with other oxidation technologies.The groundwater in Daqing area is weakly alkaline, and the experimental effect was the most ideal. The nCaO_2_ content added in this experiment was less, and the pH value of the solution before and after the experiment was small, which had little effect on the solution.

After 10 h of cumulative operation of GCW, the average removal rates of OTC in sand box aquifer are 74.2% and 83.3% under the action of O_3_ alone and nCaO_2_ in cooperation with O_3_. The highest removal rates in aquifer are 80.46%(C3) and 88.13%(C3), and the lowest removal rates are 69.31%(B5) and 76.81%(A5). The highest removal rate was only 0.5% and 0.3% higher than that of aeration for 10 h. After that, the tail concentration was 5.16 mg/L and 3.4 mg/L in the stable stage of the experiment, which indicated that nCaO_2_ combined with O_3_ enhanced the OTC degradation compared with O_3_ alone.

### The variation of OTC concentration after stopping aeration

At 0 h, 3 h, 6 h, 9 h, 12 h and 15 h after the experiment of O_3_ assisted circulation well repair technology, the average concentration of oxytetracycline in the simulated sandbox was 5.16 mg/L, 5.46 mg/L, 6.13 mg/L, 6.75 mg, 7.33 mg/L and 7.45 mg/L. Because in the aeration experiment, ozonation oxidizes part of the oxytetracycline dissolved in the aqueous phase, because the surface of the sand is rough and the specific surface area is very large, part of the oxytetracycline in the solution is adsorbed. The oxytetracycline adsorbed in the interstitial space of the sand desorbed and gradually transferred to the liquid, resulting in an obvious rebound in the concentration of oxytetracycline in the solution.

The average concentration of oxytetracycline in the sandbox was 3.4 mg/L, 3.25 mg/L, 3.12 mg/L, 3.05 mg/L, 3 mg/L and 2.96 mg/L after the repair experiment of nCaO_2_ combined with O_3_ enhanced circulating well. It can be seen that the concentration of oxytetracycline in the sandbox decreased slowly after the end of the experiment. The reason is that after the end of the experiment, the nCaO_2_ particles that remained in the sandbox did not participate in the reaction slowly released H_2_O_2_ in the water. Catalytic O_3_ molecules produce more oxidizing hydroxyl radicals, oxidize oxytetracycline in water, and spread to the whole simulation box with the flow of groundwater. The experimental results show that nCaO_2_ combined with O_3_ in-situ remediation technology can eliminate the rebound of pollutants in in-situ remediation, expand the repair area and prolong the repair time. And the pH value of the solution changed little before and after the experiment, which had little effect on the pH value of the solution.

## Conclusion

Through experiments, the effects of nCaO_2_, O_3_, nCaO_2_ and O_3_ on oxytetracycline degradation were analyzed, and a set of three-dimensional experimental device was designed to simulate the groundwater aquifer. The effects of different oxidants (nCaO_2_, O_3_, nCaO_2_ and O_3_) on oxytetracycline degradation in groundwater circulation wells were explored, and the following conclusions were obtained.

1. The effect of using nCaO_2_ enhanced circulation well alone to remove OTC from groundwater is not obvious. When using O_3_ enhanced circulation well alone to remove OTC, the OTC near the circulation well will be removed first, and the cone-shaped repair range around the center line of GCW will gradually form. The circulation of water can basically involve the whole tank, and the best time for GCW to repair OTC is 10 h, which can degrade 74.2% of pollutants in aquifer. Compared with O_3_ alone, the efficiency of nCaO_2_-O_3_-enhanced GCW in repairing OTC is obviously improved.
2. After the aeration experiment of the circulation well was carried out for 10 h, the remediation experiment entered the tailing stage. After the aeration was finished, the oxytetracycline concentration in the remediation sand box of O_3_-assisted circulation well obviously rebounded, but nCaO_2_ and O_3_-enhanced circulation well could avoid the rebound of pollutant concentration, and the oxytetracycline concentration decreased slowly. The experiment shows that nCaO_2_-O_3_-enhanced circulation well has better effect on oxytetracycline degradation than O_3_-assisted circulation well alone.

NCaO_2_/O_3_ has obvious advantages in the remediation of contaminated sites. On the one hand, the technology can select the optimal O_3_ aeration rate and nCaO_2_ dosage for different contaminated sites, and the application scenarios are very extensive; on the one hand, as an in-situ remediation technology, nCaO_2_/O_3_ technology has the advantages of low cost, consumption resistance, high operational stability and high pollutant removal efficiency, which is in line with China's national conditions, especially for large areas of non-sensitive land contaminated sites, organic steam intrusion contaminated sites and sudden environmental pollution events. The remediation effect is remarkable and the market prospect is broad. The remediation system has a certain continuity, will not interfere with the flow of groundwater, nor will it cause secondary pollution to the soil. In summary, nCaO_2_/O_3_ technology has broad application prospects in the treatment of complex contaminated sites.

## Data Availability

The datasets used and/or analysed during the current study available from the corresponding author on reasonable request.
